# Marked Mydriasis and Neuritis Nervi Optici Associated with Galactorrhea Following Citalopram Treatment: A Case Report and Discussion

**DOI:** 10.1155/2011/191735

**Published:** 2011-08-15

**Authors:** Horst J. Koch, Heike Zellmer

**Affiliations:** Department of Psychiatry and Psychotherapy, HELIOS Clinic Aue, D-08280 Aue, Germany

## Abstract

We report the case of a 25-year-old women suffering from major depression who was treated with citalopram for several weeks with doses between 20 mg and 60 mg. She gradually developed marked mydriasis within 2 months after treatment and subsequently neuritis nervi optici. Moreover, abrupt galactorrhea occurred after 2 months of treatment. All neuro-ophthalmological, neurophysiological, clinical laboratory, and neuroradiological diagnostic efforts did not reveal an underlying organic pathophysiology. The ocular symptoms disappeared rapidly after the discontinuation of citalopram and pulse therapy with methyl-prednisolone. However, galactorrhea persisted for a few weeks necessitating treatment with bromocriptine.

## 1. Introduction

Anisocoria and mydriasis are neurological symptoms which necessitate immediate medical attention including neurological, ophthalmological, and neuroradiological diagnostics. Brain stem lesions, cranial nerve damage, or even brain tumors may be possible underlying conditions. Moreover, Adie syndrome may cause mydriasis in combination with weak reflex responses and provoke diagnostic pitfalls. Anisocoria and mydriasis or related ocular findings following intake of SSRIs (selective serotonin reuptake inhibitors) are rare side effects, and only few cases are documented in scientific literature. Okzan and Kivrak [1] published the case of 24-year-old nurse who developed anisocoria after one-month treatment with sertraline. Dorell et al. [2] observed a case of diplopia after therapeutic use of citalopram. Already in 1994, Barrett published one case of anisocoria associated with administration of sertraline [3]. Obviously, anisocoria appears to be a very rare side effect occurring during clinical use of paroxetine [4] so that a class effect of SSRIs cannot be excluded. Whereas maculopathy appears to be very rare side effect during treatment with citalopram [5] dopamine-mediated galactorrhea is occasionally associated with citalopram, escitalopram and other SSRIs [6–9]. 

We present the case of a 25-year-old women who developed a trias of marked mydriasis, neuritis nervi optici, and finally galactorrhea after treatment with citalopram.

## 2. Case Report

The 25-year-old, slightly overweighted women for the first time suffered from increasing depression in summer 2008. Possible trigger factors were the work on a ward of apallic patients, a new partnership, and the move to a new apartment. In October, she consulted a psychiatrist, her psychopathology corresponding to a moderate depression (ICD 10 F32.1). Due to ineffectiveness of opipramol (maximum dose 2∗50 mg) citalopram outpatient-treatment was added with increasing doses (20 to 60 mg). Although the depression symptoms improved slightly, she was admitted to the psychiatric ward due to undulating mood in November 2008. During the interview, she showed a moderate-to-severe depression with somatic syndrome without suicidal ideation. Due to disturbed sleep, zolpidem (5 mg) was administered transiently, and after a few days after admission, mirtazapine with increasing doses (15 up to 30 mg) was initiated. 

In the neurological examination on admission she showed a marked mydriasis with normal reaction to light stimuli. Moreover, the patient complained of intermittenl paraesthesias and dysaesthesias particularly in both arms and left sided headaches. An ophthalmologist was consulted the same day and diagnosed a left-sided acute optic neuritis with paracentral scotoma. We immediately started an intravenous pulse treatment with methyl-prednisolone (initially 1 g for 3 days, then oral dose reduction step by step with 10 days). Simultaneously, neurological and cardiologic was initiated to assess inflammatory or ischemic brain disorders within 5 days. Thorough electrophysiological investigations including visual evoked potentials, cMRT, clinical laboratory, and cerebral spinal fluid analysis did not indicate an inflammatory brain disorder or ischemia. Blood coagulation tests were within normal range. Neurosonography showed physiological extra- and intracranial vessels. Echocardiographic investigations (TTE and TEE) confirmed a very small PFO which had probably no clinical relevance, but low-dose Aspirin (100 mg) was recommended. Gynecological assessment revealed normal findings apart from left sided lactation; prolactin was not increased.

The patient responded well to steroid pulse therapy but temporarily tended towards increased blood glucose levels and ankle edema occurred. Blood glucose normalized after cessation of steroid treatment and edema disappeared after small doses of frusemide.

## 3. Discussion and Conclusion

The patient presented is the first case in the literature suffering simultaneously from the trias of marked mydriasis, optic neuritis, and galactorrhea. The exact mechanism of action remains speculative, but some effects have been already observed experimentally or clinically. Mydriasis obviously can be explained via 5-HT1a-receptor stimulation [10]. Optic neuritis may be caused by several drugs such as isoniazid or ethambutol, but only in very rare cases following administration of SSRIs [11]. Acute allergic reactions occurred after the administration of escitalopram and may, therefore, serve as a suitable pathophysiological model although the course was much more delayed and less severe in our patient [12]. Experimental data using arthritis models in rats discuss a proinflammatory role of central serotonin which indicates an aggravating effect of SSRIs with regard to the inflammation cascade [13]. This experimental findings were only partially confirmed by population-based determinations of C-reactive protein (CRP), which revealed increased CRP levels only after use of tricyclic antidepressants but not after intake of SSRIs [14]. However, the results are equivocal, as fluoxetine obviously ameliorates neuropathic or inflammatory pain conditions [15]. 

Mydriasis, possibly due to brain activating properties of serotonin, has been confirmed in healthy volunteers [16]. Galactorrhea occurred in patients during treatment tricyclics (imipramine) or SSRI (escitalopram) without depending on increased prolactin levels [17]. Euprolactinemic galactorrhea is discussed as the result of indirect inhibition of tuberoinfundibular dopaminergic neurons. On the contrary, hyperprolactinemia and subsequent galactorrhea has also been observed during treatment with citalopram [8].

In conclusion, the exact underlying toxicological mechanism of optic neuritis in our patient, however, remains to be elucidated. With regard to galactorrhea, altered hypothalamic dopamine availability may be responsible for the side effect. A general CNS-stimulating effect of serotonin may be the cause of mydriasis following intake of SSRIs. Ocular and endocrinological symptoms only rarely occur during treatment with SSRIs but should be taken into consideration should the patient have corresponding complaints.
